# Attentional skills following serious traumatic cervical spine injuries in young sports men

**DOI:** 10.1192/j.eurpsy.2025.2016

**Published:** 2025-08-26

**Authors:** ?. Syrmos

**Affiliations:** Aristotle University, Thessaloniki, Greece

## Abstract

**Introduction:**

Cervical spine injuries are serious traumatic situations

**Objectives:**

Aim-Aim of this study was to evaluate the attentional skills in cases with serious cervical spine injuries, in young sports men.

**Methods:**

Material and Methods-10 young male amateur sports men (1 soccer player, 1 basketball player, 1 volley-ball player, 1 hand ball player, 1 boxer, 1 long runner, 1 fast runner and 3 swimmers) were participated in this study, 20 days more or little less after serious cervical spine injuries. Range of age 19-39 years and mean age 29 .We used specific performance tests, letter cancellation exam, naming trials and iq tests.

**Results:**

Results-All of them(10,100%) reported deficits in all areas of attention function and also cognitive and emotional status.9 of them(90%) reported also difficulty in complex task activities, especially when time limits were imposed.

**Conclusions:**

Conclusions-All these exams are good and efficient tools in order to evaluate this kind of patients.

**Disclosure of Interest:**

None Declared

